# “We’re Lucky to Have Doctors at All”; A Qualitative Exploration of Australian Farmers’ Barriers and Facilitators to Health-Related Help-Seeking

**DOI:** 10.3390/ijerph191711075

**Published:** 2022-09-04

**Authors:** Melissa J. Hull, Kate M. Gunn, Ashleigh E. Smith, Martin Jones, James Dollman

**Affiliations:** 1 Alliance for Research in Exercise, Nutrition and Activity (ARENA) Research Concentration, Allied Health & Human Performance, University of South Australia, Adelaide, SA 5000, Australia; 2 Department of Rural Health, Allied Health and Human Performance, University of South Australia, Adelaide, SA 5000, Australia; 3 School of Nursing & Midwifery, La Trobe University, Bundoora, Melbourne, VIC 3083, Australia

**Keywords:** rural, help-seeking, agriculture, health, mental health, barrier, farm

## Abstract

This study aimed to explore barriers and facilitators that impact on farmers’ help-seeking behaviours for health and mental health concerns. Fifteen semi-structured interviews were conducted with farmers (12 male; age 51.7 ± 12.6 years) from three rural regions in South Australia. Interviews explored demographic and farm-related characteristics, perceptions of individual (and where relevant family) health and mental health concerns and experiences, and perceived barriers of health support-seeking. Thematic analysis was used to identify key themes. Four key themes were identified relating to help-seeking; personal attitudes and beliefs, farm-related barriers, health system barriers and the provision of support from family and friends. Dominant personal attitudes included valuing independence, strength and privacy. Farm related barriers included the ‘farm comes first’ and the fact that ‘farm work is never done’. Health system barriers included issues relating to availability of choice and access, professionals (lack of) understanding of farm life, and time and financial costs of accessing care. Provision of support from family and friends involved informal help and advice, including facilitating access to professional support. Multiple attitudinal, structural, and farm-related issues affect farmers’ help-seeking. Professionals who understand farm work practices and routines are valued by farmers and this is likely to facilitate access to care. Workforce development programs and community programs that involve farmers’ perspectives as consumers and co-designers, using evidence-based strategies, may assist in strengthening these relationships.

## 1. Introduction

Agriculture is consistently recognised among the most dangerous occupations for workplace injury and mortality, both in Australia and internationally [1,2,3,4]. Farmers are regularly exposed to a range of psychosocial, physical hazards and choices due to the isolated nature of farm work and the many factors beyond their control that affect their livelihood, which can significantly affect their health, mental health and behaviour [5]. Of concern is the fact that farmers in Australia and other developed countries experience a disproportionate burden of suicide, illness, injury and premature mortality [6,7,8]. Australian farmers have been found to have relatively high rates of prostate and blood cancers, transport accidents and cardiovascular disease compared to their non-farming rural counterparts [9,10,11,12,13]. While these health challenges are likely multifactorial in nature, these issues may reflect a wider problem whereby farmers face barriers to responding to and addressing symptoms in a timely manner.

Australian farmers are half as likely to have visited a general practitioner (GP) or mental health worker in the previous 12 months compared to their non-farming rural counterparts [14], underscoring the urgent need to address this public health challenge. These issues are likely to be compounded by an inability to access appropriately trained health and mental health care providers, and other attitudinal factors more prevalent among farmers [15,16]. When combined with the risks associated with living and working in rural and remote areas more generally, including prevalent risk taking behaviour, high rates of alcohol and drug use, the need to travel long distances on roads that are more often poorly maintained and farmers’ very high workloads, it is unsurprising that farmers have worse health outcomes, and often view their health through a functional lens [17,18,19].

The ‘agrarian myth’, which idolises farming as healthy and stress-free is a contentious issue, given the wider research that confirms the range of health disparities that exist between Australian farmers and their non-farming rural counterparts [20]. The perpetuation of this myth, along with the values of self-sacrifice and hard work in contemporary Australian farming structures have also been linked by sociologists, to low-self worth, shame and male famers’ elevated suicide risk [21].

Previous research has shown that Australian farmers identify strongly with the idea that adequate health is defined by one’s ability to complete daily activities on the farm [19,22]. Farmers in the USA have expressed similar attitudinal barriers towards help-seeking, as well a lack of trust of ‘outside’ health professionals [23].

Previous quantitative research has shown that Australian farmers, compared to other rural people, face barriers to help-seeking for mental health related issues including a need to remain stoic, self-reliant and in control of the situation, as well as a lack of understanding of their treating health professional [15]. Qualitative exploration of these issues in Australian farming populations is somewhat more limited. Emerging research suggests lifestyle factors, the unpredictable challenges of farm life and resource pressures including time and finances, influence mental health help-seeking of Australian farmers [24,25,26]. However, little attention has been devoted to determining other barriers and considerations that may affect general help-seeking across the range of health issues, in this at-risk farmer group. Accordingly, the purpose of this study was to provide an in-depth exploration of the broader individual, social and systemic factors that influence physical and mental health-related help-seeking from the perspective of Australian farmers.

## 2. Materials and Methods

An in-depth qualitative descriptive design was employed [27,28]. Semi-structured interviews were completed either in person or via telephone (M.J.H.). An interview guide was developed following a wider literature review, expert opinions from the authors and considering the results of a cross-sectional study identifying barriers to mental help-seeking across farming and non-farming rural populations [15]. The resultant interview guide was piloted (before the full interviews were conducted) with two rural men to assess question length, language, and general acceptability of the questions. Interview topics included: personal background and general farm information; perceptions of personal health (and, where appropriate, the family’s health); experiences and barriers to physical health support-seeking; experiences and barriers to mental health support-seeking; and experiences of sleep, fatigue and injury. Language was amended slightly following the piloting process. In person interviews were held at a neutral community facility, in a private room. The reporting of this study has been reported considering the consolidated criteria for reporting qualitative research (COREQ) guidelines [29].

### 2.1. Participants

Participants met the inclusion criteria if they were:

1. Over 18 years of age;
2. Owned or worked on a farm to produce goods for sale at a commercial level;
3. Lived or worked in any of the three target regions of South Australia;
4. Did not report any cognitive impairment that might inhibit their ability to answer questions.

The three regions of South Australia selected for this study were the Eyre Peninsula (EP), Mid North (MN) and the South East (SE). These regions range from outer regional to very remote based on the Australian Bureau of Statistics [30,31,32] remoteness structure. They also comprise a wide variety of farming types including livestock production (e.g., beef cattle, sheep and lamb), cereal and broadacre cropping, dairy farming and grape growing. The regions have varied levels of physical access to health and mental health services.

### 2.2. Recruitment

A purposive sampling strategy was used to seek representation from participants across a range of regions, age groups, genders and levels of farm experience. Participants were recruited via multiple methods including advertisements and stories in regional news outlets (print and radio), flyers in local businesses and public places, email distribution lists from agricultural bureaus, social media accounts and the authors’ personal contacts. Ethical approval for the study was received from the University of South Australia’s Human Research Ethics Committee (protocol # 0000033469).

Prior to the interview commencing, participants provided full written consent, including to be audio recorded. Interviews ranged from 30 to 90 minutes. Using a reflexive, semi-structured approach, interviews continued where possible until a rich, complex representation was collected from across the three regions [33]. Recordings were transcribed verbatim and participants were given the opportunity to verify their transcripts. Most declined this opportunity citing time constraints. One participant did return an adjusted transcript with the only change clarifying a technical term.

### 2.3. Analysis

A preliminary data analysis allowed M.J.H. to compare the verified transcripts with the field notes and identify any immediate issues that may require further clarification from participants [34]. Braun and Clarke’s method of thematic analysis was then used to identify the themes that were generated from the data [33,35]. Data were managed using NVivo data management software (Versions 10 & 11; QSR International, Burlington, MA, USA).

Recordings and field notes were initially transcribed and read in full to ensure familiarisation with the data [33]. Initial codes were generated and an inductive or ‘bottom-up’ approach was employed, with the codes and themes generated by the data rather than an existing theoretical framework. A subsample of coding was conducted independently by M.J.H. and K.M.G. before being discussed in detail. The remaining coding and initial drafting of themes were conducted by M.J.H. Subsequently, K.M.G. reviewed all themes, which were refined with M.J.H. via discussion, until full agreement was reached.

### 2.4. Reflexivity

At the time of data collection, M.J.H. was a novice qualitative researcher who had undertaken intensive training in qualitative approaches prior to the start of these interviews. As a female in her mid-20s M.J.H. grew up in a rural farming community with multiple family friends who were farmers. M.J.H. conducted all interviews and was the primary lead on the analysis.

K.M.G. is a clinical psychologist and behavioural scientist. K.M.G. brings significant experience in rural health and farmer mental health research and lived experience from living on a family farm. She provided detailed qualitative support during this research, was consulted on all stages of the project and assisted with coding and with describing/naming the themes identified via thematic analysis.

M.J. has previously worked in the UK, but since arriving in Australia has split his time between rural and metropolitan South Australia investigating the best practices in health workforce development for rural Australia.

J.D. has worked extensively with rural populations particularly those focused on physical activity and cardiometabolic health outcomes. He has lived and worked in regional SA for over 10 years and regularly travels across regions to maintain his connections with the community.

A.E.S. spent her childhood in both rural and urban communities across Queensland (Toowoomba and Brisbane) and South Australia (Adelaide Hills). She brings lived experience of farm and metropolitan living.

All authors share a positive view of living in rural regions having lived in regional areas for many years and appreciate the value that agriculture has in these regions. To mitigate bias M.J.H. kept a research diary before and after all interviews, and throughout the analysis. She regularly discussed and debriefed interviews with J.D. and K.M.G. during data collection and throughout analysis to highlight and mitigate the impact of any subconscious bias that may have influenced data collection and analysis.

## 3. Results

### 3.1. Demographic Characteristics

In total, 15 interviews were conducted with farmers (12 male and three female, range 27–67 years). Participants were involved in a variety of farming pursuits, including beef cattle production, sheep and lamb production, dairy, cereal and broadacre cropping and hay or feed production. The demographic characteristics of the sample are provided in Table 1.

### 3.2. Thematic Analysis

**Figure 1:**
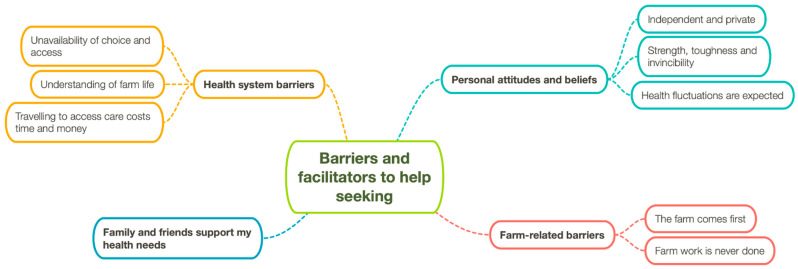
Thematic network diagram representing overarching themes and subthemes.

Four key themes relating to farmers’ help-seeking behaviour were identified from the data. These included personal attitudes and beliefs towards accessing health care, farm-related barriers, health-system barriers and the facilitating role of family and friends in supporting health needs. Sub-themes were also identified and are detailed below. Following a further review of codes and definitions, thematic network diagrams were drawn, based on Attride-Stirling’s work [36], to assist in visualising and reporting themes and interrelationships (Figure 1). The extracts included focus on the clearest points made by participants in relation to each theme.

#### 3.2.1. Theme 1: Personal Attitudes and Beliefs

Throughout the interview, process participants were asked to recall and reflect on their experiences of seeking help for physical or mental health issues. Key to these experiences were the varied attitudes and beliefs that were held across the participant group. These attitudes and beliefs were considered barriers to help seeking for the participants. Unless clearly stated, no distinction was made between seeking help for physical or mental health related issues by most participants. Combined, these attitudes and beliefs indicated participants did acknowledge a variety of health issues that impacted them. However, they were generally less likely to look to seek help for these issues unless prompted by a significant other, or in a time of crisis.

##### Subtheme 1: Independent and Private

The main discussion regarding participant’s attitudes and beliefs to help-seeking focused on individual’s preference to maintain their independence and privacy. Many viewed this sense of independence as being central to their ability to continue their ongoing farm work and work as independent problem solvers for any challenges that arose on the farm. As one participant discussed:

“I’m just the sorta person that thinks that you’ve gotta, you gotta set to the task and fix the problem... generally the best person to do that’s yourself because mentally there’s a sort of defeat, fronting up to someone.”

(Male, mid 40s #1, SE)

Many participants were forthcoming when disclosing health conditions when directly asked about their self-reported health. However, when considering how they preferred to use health services they indicated a strong preference and normalised belief that keeping problems to themselves (or within their immediate family) and maintaining privacy was best.

“I’m on medication [for depression] but you know it doesn’t worry me; I don’t advertise the fact that I try … and hide it, you know.”

(Male, late 60s, EP)

“People say that they’ve gotta go to the chiropractor, but they won’t tell you they’ve gotta go to the counsellor.”

(Male, mid 40s #2, SE)

Unsurprisingly this was strongest in relation to those who had experienced mental health concerns. One participant who had experienced a previous acute mental health crisis reported a strong internal need at the time to hide their emotions and feelings from close family.

“When I was acutely..., I was probably ashamed of my depression. I tried never to show my true, total emotion in front of my wife. I tried to do that in private.”

(Male, mid 50s, MN)

##### Subtheme 2: Strength, Toughness and Invincibility

Closely related to independence was the notion that one needs to be ‘strong’ and ‘tough, to maintain face like a man’. To a certain extent, male participants discussed these issues when reflecting on risky solutions they had used to solve farm related problems. It was only after the fact that they were able to reflect on how dangerous that solution could have been. While these situations were commonly acknowledged across participants the wider need to take risks (in relation to physical injury) to get things done was highlighted by one South East farmer:

“You need a certain amount of resilience and hardiness.”

(Male, mid 40s #2, SE)

Discussions with female participants highlighted these differing expectations. While participants were asked to reflect on their own help-seeking, female participants often spoke of their male partners’ reluctance to show ‘weakness’ or ‘hurt’, which they (males) believe accessing health care services would reveal:

“I think it’s built into their character, that farmers are pretty capable, tough, don’t need help, can be independent and can work everything out themselves, which is a big barrier to get over.”

(Female, mid 60s, EP)

Male participants also shared this view, although this linked back to their preference to be independent in their problem solving and wish to continue preserving until it was no longer possible. Again, these views intersected with the earlier attitudes towards being independent in their care and work. When asked how or why they viewed their health in this way, participants commonly returned to the idea of health as a problem to be solved, where if they knew the (individualised) problem then they should be able to logically work through a solution.

“I felt like it was a little bit of a cop-out that I went and saw him [the GP]. I knew what the problem was, I just had to fix it.”

(Male, mid 40s #1, SE)

“We sorta tend to tough it out a bit longer.”

(Male, mid 40s, MN)

This wish to maintain an aura of being strong and tough was also commonly followed by reference to being invincible. Invincibility was considered in relation to being indestructible. Participants discussed this barrier being something one had to almost grow out of. One participant reflected on their years of experience and acknowledged how with age and experience their views had changed:

“Well, I’m still active, I realise I’m not invincible, and I’m starting to look after myself a bit better I think.”

(Male, mid 60s, EP)

This was another prompt where female participants reported seeing this attitude play out in the behaviours of their male partners. It was interesting to note that these women did not acknowledge the same traits in themselves:

“They just really think that they can keep fighting on and that nothing can really happen to them.”

(Female, mid 30s, EP)

When discussing these attitudes and experiences it was clear that many viewed these traits of strength, toughness, and continued perseverance as part of what made them successful at farming, and as such met the occupational needs of the profession.

##### Subtheme 3: Health Fluctuations Are Expected

When asked about how they defined health issues, participants were quick to identify that they expected to experience fluctuating health daily. Most commonly, this was operationalised as the experience and presence of some pain was entirely normalised in their industry. As a result, participants reported normalised behaviours including largely ignoring any sense of pain or health condition, until either someone else noticed the issue (indicating it had worsened to an obvious level), or it reduced or stopped their ability to complete their daily work.

“Got a crook back that plays up all the time… But that’s just ongoing; it’s not debilitating, but if it goes out, it’s pretty ordinary for a few days, and you get it right.”

(Male, early 60s, EP)

This view of health and expectations for pain and regular and recurrent daily injury was clearly viewed differently by most participants who had grown to expect a different ache or twinge. Participants reported a wide range of minor (yet painful) injuries that were an almost daily experience for them. These ranged from encounters with spooked and unpredictable livestock through to missing a step on a tractor resulting in a jarred leg.

“I dislocated a couple of fingers there yesterday, and I don’t view them as injuries. They’re just part of it, it’s just what happens.”

(Male, mid 40s #1, SE)

“It’s just one of those things; you live in a constant area of pain.”

(Male, late 50s, SE)

Participants were quick to agree that they preferred to ignore health problems, at least initially before they decided whether to seek help. Closely associated with this was the idea that one should not focus on the fact that one is sick or feeling unwell, and that it is preferable to ignore it, at least for a while:

“Well, why wouldn’t ya? Nothing to do by sitting around doing nothing. Might as well keep going, yeah.”

(Male, late 50s, SE)

“I don’t go unless I’ve got something wrong.”

(Male, late 60s, EP)

The main exception was if participants had been previously diagnosed with a serious health problem (e.g., cancer, heart disease or a significant episode of depression or anxiety). Understandably, the strategy to ignore symptoms in these cases was not as highly valued or endorsed:

“I thought it was very suspicious. I got very sweaty and things started to go a bit pear-shaped.”

(Male, late 60s, EP)

“You’re getting around that network, and yeah, I won’t sweep it under the carpet I’ll talk about it.”

(Male, mid 40s #1, SE)

Closely linked with this ‘wait and see’ approach, was the belief that participants did not wish to complain about their health issues. Participants were wary of being viewed as weak, and in some cases were concerned about wasting limited health resources in their area, taking an appointment that they may not have needed. Interestingly, some also conceded that they were more likely to complain regarding less serious illness (e.g., colds and flu) compared to a problem they perceived was more serious (e.g., cancer, heart disease).

“I’m going to react when I have a bad flu. I’m going to lay on the couch and try and convince everybody that I’m very close to death and I’m happy to tell anyone that walks past that I’m only a few breaths away.”

(Male, mid 40s #1, SE- in jest)

Regardless of the specific situation, participants largely agreed that they viewed their ability to manage these health fluctuations (and deciding on the severity of the illness or injury) by their ability to continue on with farm work. Continuing with some manner of work, whether it was an easier physical task, or even just office-based bookwork, participants had a clear preference to continue with some form of productive work. Medical attention was only sought if work was no longer possible.

“Unless you’re feeling really, really crook, but even that you always find something to do … don’t go sitting in the house all day long, no.”

(Male, mid 50s, SE)

“You know if you’re feeling flat you might do any easier job, but there’s no days off, ‘cos you live and work at home.”

(Male, mid 40s #3, SE)

#### 3.2.2. Theme 2: Farm-Related Barriers

As mentioned previously, participants strongly endorsed several farm related issues that delayed or stopped their help seeking. All participants agreed that the all-encompassing nature of farm work, and the numerous unpredictable factors that can affect the timing of tasks at times made seeking appropriate help for any health concerns a secondary priority to the overall health and success of the farm. The prioritisation of the farm having to come first was clearly explained by each participant relevant to their family, social and individual circumstances.

##### Subtheme 1: The Farm Comes First

The enormity and demand of farm work was raised by everyone when asked how the farm impacts on their help seeking. Participants acknowledged their sense of ongoing responsibility to provide economic and lifestyle stability for their family, and associated economic and social benefits for their region as a key barrier for accessing health services.

“I s’pose the other thing we get in a small community is there’s a lot of pressure to I s’pose provide for your community… There’s just a lota community work to do in a small community and I s’pose that’s something at times just increases your workload but someone’s gotta do it.”

(Male, mid 40s, MN)

Linked to this was the normalised beliefs and expectations that their health would likely fluctuate day to day, and during busy times (such as seeding, or shearing) these fluctuations would be more easily ignored in preference to the ongoing maintenance of the farm.

“Our crop must go in on time; everything’s gotta be right because you know two bad years in a row and you can go from being financially okay to being in deep, deep trouble.”

(Male, mid 60s, EP)

So, health wise how many farmers will ignore health-warning signals because of other pressures, especially financial pressures? I’d say a huge amount, and I have also been guilty of the same sin.”

(Male, mid 50s, MN)

For those who had sought help for health problems they reported challenges with accessing specialist services. Commonly participants reported they had been unable to make appointments, or comply with ongoing treatment recommendations due to the ongoing farm work, and increased pressure from seasonal tasks.

“But to get that organised, to leave a farm and get everything set-up so that everything keeps going while you’re away? I’ve never achieved it. That’s the sort of thing I think we do miss out on because there’s too much ongoing stuff on farms.”

(Female, mid 60s, EP)

Participants also reported issues in finding replacement workers and needing to gain extra support to keep the farm running safely. Managing a reasonable level of risk was also considered important to ensure the farm could continue to operate and was seen as interlinked with the previously discussed attitudes surrounding strength and invincibility.

“I believe most farmers are under manned to what they should be. I think we’re all short staffed and people are always in a hurry and that’s what makes people get tired, overtired and still work and that’s where the danger comes in.”

(Male, mid 50s, EP)

Some participants discussed how they had worked to integrate their treatment regime into their daily farm life:

“So it might mean that morning I don’t do Pilates but I’ve got it in the back of my mind that I can stop at any time during the day and do it. It doesn’t have to be done in the house, it doesn’t have to be done in the morning.”

(Male, mid-60s MN)

##### Subtheme 2: Farm Work Is Never Done

The ongoing nature of farm work and having a never-ending list of tasks was highlighted by most participants as a further barrier to their help-seeking. Combined with their general lack of concern over many minor or early symptoms of ill health this workload was reported to lead to delay in help-seeking as the weeks and routine tasks of farm life tend to blur together.

“Unfortunately, in our game you just get stuck there, and you just do seven days a week and one week goes into another.”

(Male, mid 40s #1, SE)

“If there’s work to be done, you tend to … I’ve never come to this situation where we’ve had something important on and I haven’t been able to do it.”

(Male, early 60s, EP)

As previously mentioned, participants were quick to raise their need to feel productive in whatever farm related tasks, they were capable of doing (despite how unwell they felt). The circumstances of all participants, who lived on their farms also appeared to lead to this perception of needing to remain busy and engaged, with bookwork often waiting for participants when they returned to the main house.

“If we’re busy we just don’t have a choice, you’ve just got to be busy.”

(Male, early 40s, MN)

#### 3.2.3. Theme 3: Health System Barriers

Participants reported numerous structural barriers related to the health system that impacted their help-seeking. These issues mirror those of rural Australia generally, however participants reported a range of related challenges that were somewhat unique to farming in rural and remote areas of Australia.

##### Subtheme 1: Unavailability of Choice and Access

The main issue most participants reported when asked how they found their local services related to the accessibility of the service and choice of practitioners. Accessibility of the service was viewed both in terms of physical distance/travel time to and from the service as well as the range of appointment times that were available, and any consequent waiting time. In these discussions participants commonly referred more to the impacts this had on their families rather than themselves as individuals, as we had already established most did not seek routine or regular care for themselves. Most of these challenges were discussed primarily in relation to physical preventative health, but the challenges for those who had experience of seeking mental health support were particularly apparent:

“Where we live, we are at least half an hour to 45 minutes from any convenience whatsoever. That can be trying, particularly when you’re raising a small family.”

(Male, mid 40s #2, SE)

“So, there’s sorta go to Adelaide if you need, but there’s really not a lot of support services locally.”

(Male, mid 40s, MN)

The female participants in this study described significant challenges with being able to see a female doctor. While the gender of practitioner was less of a concern for the male participants, they shared similar concerns relating to having any choice of practitioner within their local region. Linked to these challenges was the perception of trust and confidence in local services. Trust in their local service appeared to be impacted by the rotation of doctors throughout various regional services. This sense of distrust was clearly linked to community word of mouth, with participants reporting stories whereby negative word of mouth, or poor prior experience they or family members had faced were discouraging to their ongoing help-seeking:

“There’s doctors in [regional centre] that I will never go to, and that’s word of mouth, and not word of mouth from one person; it’s just general from a lot of people.”

(Male, mid 60s, MN)

“Usually, the newer ones come in; people don’t know the new ones… Get the old reliable one you’ve dealt with before, so you try and use him if you can.”

(Male, mid 50s, SE)

Frustration with long waiting times, and appointments that did not keep to time was obvious for most participants. This combined with ongoing demands of farm work compounded participants reluctance to seek help for routine or preventative care once they had booked an appointment:

“Not one particular doctor but it’s 40 minutes from where we live, and I’m not exaggerating; if I want to see a particular doctor, I will have to wait three months. Now that’s frustrating.”

(Male, mid 40s #2, SE)

“You can’t go to a doctor’s surgery when you’re in business and have your own appointments and spend two hours sitting in the waiting room.”

(Male, mid 40s #3, SE)

##### Subtheme 2: Understanding of Farm Life

Once participants were able to overcome the barriers to get an appointment, they commonly reflected on ongoing challenges of building rapport and a trusting relationship with their health care professional. Finding a health professional who had some understanding of farming or agricultural practices and community values was important for participants. As was the ability for that professional to communicate in a manner that the participant could understand. Ongoing challenges were reported including poor communication, negative experiences with locum doctors (i.e., doctors who provide short-term coverage while permanent staff are unavailable), and an inability of professionals to understand the rigours and challenges of farm life:

“You know, I’m sure that people, health professionals in the city, think we are mad. They really do.”

(Male, mid 40s #1, SE)

“Overseas-trained doctors, well, I was going to say, doctors, medical people. Because sometimes they are difficult to understand and maybe they find me difficult to understand, so communication can be a problem.”

(Male, mid 60s, MN)

##### Subtheme 3: Travelling to Access Care Costs Time and Money

Given the challenges that participants faced while trying to access care in their home community or region, it was not surprising that many expressed a preference to access health care from a larger regional or metropolitan area. Although participants acknowledged this choice relied upon them successfully finding cover to ensure their farm work would continue:

“Then it might also depend on whether I can access somebody to do that work instead of myself.”

(Male, mid 60s, MN)

When asked to explain their preference, participants reported being offered shorter waiting times for appointments, and a perception of improved quality of service and speed of diagnosis. Participants also viewed these longer trips as a more time efficient method of completing off-farm work, whereby they would prefer to link appointments or tasks to be done in town to increase the worth of the trip.

“I’m not going to drive to [regional centre] for one appointment. I like to be a bit more efficient than that. So, there’s probably more efficiency in heading to [major city], but I’ll still want to work it in with a few other jobs.”

(Male, mid 40s #2, SE)

“I think most of the services are down here; it’s just the waiting, the cost and the travel.”

(Male, mid 40s, SE)

The time and money required to travel to access these health care preferences was also highlighted by participants. In many cases, participants reflected that family and friends assisted with accommodation away from home or word of mouth in recommending certain health care providers. One participant summarised this perspective after needing to seek urgent treatment in Adelaide (metropolitan centre) for an injury affecting their child:

“All the costs, too. Like, having to come over because we haven’t got the facilities over home. So, that’s where the cost factor comes in too. You know, we’re lucky we’ve got somewhere to stay here in Adelaide, but if you haven’t … If you had to pay for accommodation plus treatments and all of that, it soon adds up.”

(Female, mid 40s, EP)

#### 3.2.4. Theme 4: Family and Friends Support My Health Needs

Participants reported a preference for lay support (or informal support provided by family and friends) rather than directly seeking formal and professional support for mental health concerns in particular. While all participants acknowledged mental health services were necessary to help individuals deal with some challenges, they were hesitant to acknowledge that they may need to use them themselves. In most cases, individuals were able to recount experiences of friends or family members who had sought help, but there was a clear reluctance to seek help personally should they find themselves in a similar position. This appeared connected to the earlier attitudes where independence, privacy and self-help were valued:

“While I’m a firm believer in medical intervention for people that are suffering I also believe that the problems that you resolve yourself are better resolved than the ones that are covered up by medication.”

(Male, mid 40s #1, SE)

Male participants mentioned the importance of partners acting as ‘gatekeepers’ to health care. They described a range of methods women used to facilitate such access, including booking appointments, managing medication and ‘debriefing’ after a long day:

“Their wife will most likely make the appointment and say I’ve made you a doctor’s appointment for 2 o’clock on Thursday and he says yeah fine. May go, may not go.”

(Male, mid 40s #1, SE)

“Well, it’s about them trying to get help for a start or get their partner getting them help.”

(Male, early 60s, EP)

Debriefing with friends was a valued connection that many participants reported using for additional support during times of high stress, or when they felt unable or unwilling to raise problems directly with their partner.

“Yeah I’ve got a couple of mates that I’ll ring up. It’s not really to talk about the problems but just to say ‘g’day, see how you’re going.”

(Male, mid 60s, MN)

“The men walking around on the golf course, and you know they might have just talked about... You know they could joke about it, moan and groan with each other and I just think a lot of communication and that’s good when everyone’s sort of in that same situation.”

(Female, mid 30s, EP)

The ability for female partners to provide reassurance to their partner was also considered important in breaking down some of the previously discussed barriers regarding privacy. As discussed earlier the participants were also much more likely to respond to a symptom when someone else noticed and questioned them.

“I guess if you’re down for any length of time, ‘cos you’d know your wife’d tell you.”

(Male, mid 40s #2, SE)

“If you can have open communication with a partner and support each other and recognise and have the awareness to recognise that then you can do something about it; nip it in the bud.”

(Male, mid 40s #1, SE)

The male partner clearly valued the input of their female partners in this regard to identify, communicate, and help with managing the treatment or time off they needed (regardless of mental or physical health needs). However, female partners often expressed frustration at the delayed help-seeking of their partner:

“There are times when you know very well that they should be at the doctor and they wait until it’s a lot worse before they go and that has consequences.”

(Female, mid 60s, EP)

## 4. Discussion

This study explored the factors that farmers report impacting on their ability to seek help for health and mental health-related issues. Four broad themes were identified: personal attitudes and beliefs, farm-related barriers, health-system barriers and family and friends support of their health needs.

Farmers tended to discuss health and help-seeking within an ecological framework that included personal, social (family and friends) and community contexts. In relation to farming specifically, these contexts are likely to overlap considerably given the integral role farming and agriculture plays in rural communities. This is important to consider, given that the help-seeking literature (drawn mainly from mental health help-seeking) views behavioural intention as a key factor to realising actual service utilisation [37]. A wide range of factors can influence one’s intention for help-seeking, and these are not limited to personal life [38,39]. Given the likelihood of participants experiencing closer social networks within their rural communities, it is not surprising that many of the reported stories revealed attitudes or events that originated from the experience of a friend or family member [40,41]. As such, practitioners should remember the ‘rural grapevine’ and broader community experiences often strongly influence individuals’ intentions to seek help.

A dominant theme was the role that personal attitudes and beliefs play in shaping help-seeking for health among farmers. This is consistent with previous research among both rural and urban populations. Negative attitudes and restrictive beliefs are well documented barriers that have impeded individuals (often including those living with health conditions) from accessing health care [42,43,44,45]. Previously reported attitudes among farmers and rural populations in general [19,25,26,45,46,47,48,49] were confirmed in the current study, including valuing independence, remaining ‘tough’, feeling invincible, stoicism, and a general reluctance to seek timely support due to competing interests of farm work.

Consistent with the work of Rawolle and colleagues [19] and Vayro et al. [25,26], several participants in the current study expressed the view that health concerns were not problematic until the capacity to work was markedly reduced. Participants described situations in which they had ignored their health or symptoms for a period in response to ongoing farm needs. It is possible that farmers embrace this view more than their non-farming rural counterparts because of their relative isolation and more limited interactions with broader social networks [50]. There are also financial challenges associated with soliciting support from others to effectively ‘backfill’ often highly specialised farm roles, if treatment for health issues requires time off or a period of reduced functional capacity. Replacement workers may also not be readily available in local regions as the agricultural workforce has struggled with both acute and chronic shortages and overall loss of workers away from the industry since 2000 [51]. The competing demands of farm work and health care were particularly evident during peak seasons such as harvest and seeding, when the prioritisation of maintaining the farm enterprise is driven by external factors beyond farmers’ control, such as the weather. Together with the expressed view that ‘work was never completed on the farm’, these findings are in accord with data from the Australian Bureau of Statistics (ABS) that reveal farmers work longer hours on average than others employed in non-farming occupations. In fact, over 50% of farmers reported working more than 49 hours each week in 2011, compared to just 17% of workers from other occupations [52].

Some health system characteristics represented barriers in this study. These included unreasonable waiting times for simple appointments and challenges with communicating and relating to health service providers. These factors have been reported elsewhere and relate to a perceived lack of understanding of farm life among ‘outsiders’ who do not appreciate that farming is more than an occupation but a lifestyle [26]. Participants also commonly expressed a preference to seek health related support in larger metropolitan centres due to their positive perceptions of the quality and access of treatment there. These structural health system barriers are commonly reported in the literature and while not unique to farmers, may disproportionality affect rural populations due to the necessity to travel to access these services [53,54,55].

In the light of low awareness of the capacity of services to support mental health in farming communities, participants in the current study indicated a preference to seek lay opinions from friends or family members, thereby potentially promulgating ineffective strategies. Importantly, men readily identified women as key drivers of their help-seeking behaviours, through both formal support (i.e., booking appointments or arranging medications), and more informal support as a ‘sounding board’ or supportive listener. Male farmers also indicated the important role of close friends and wider family support to help them manage situations on the farm that induce feelings of stress or anxiety. In contrast female participants all acknowledged a strong need to seek support to manage mood or anxiety issues. These attitudes are consistent with the findings of Judd, Komiti and Jackson [48] and Fennell and colleagues [43] who reported more positive mental help-seeking attitudes in females compared to males in rural populations. The differences in help-seeking between male and female participants may also be explained by the wider literature discussing traditional and cultural definitions of farming and masculinity which have been underpinned by a moral self-worth surrounding farming as a noble profession [21].

Lack of knowledge about mental health support services was not raised by participants. It is likely that this may be due to a lack of personal experience with mental health services. Those who did disclose instances of depression or anxiety discussed relying on their GP for treatment rather than having access to specialist providers such as psychologists or psychiatrists [53]. In accord, a study in rural Queensland found the majority of participants could identify the symptoms of depression but struggled to name relevant local services [56]. In the light of low awareness of the capacity of services to support mental health, participants in the current study indicated a preference to seek lay opinions from friends or family members, thereby potentially promulgating ineffective and risky strategies. Importantly, men readily identified women as key drivers of their help-seeking behaviours, through both formal support (i.e., booking appointments or arranging medications), and more informal support as a ‘sounding board’ or supportive listener.

This study has highlighted farmer-specific barriers that influence help-seeking behaviours for both mental and physical health in Australia. While research has previously identified gaps in referral practices or adherence to suggested health checks [20], this study reports, from farmers’ own experiences, the barriers they face when considering engaging with health services. There are limitations of this study that should be acknowledged: While both male and female farmers of different ages and farming regions/experiences were purposively sampled for inclusion in this study, there was an under-representation of younger male farmers. While this is a limitation in the current study, it does reflect the wider demographics of the Australian farming workforce who are on average older than those working in most other occupations [57]. Further, it is likely that younger male farmers tend to perceive themselves as healthy and therefore not in need of health services, thereby minimising the relevance of the study to them. Notably, previous health research has identified young adult males as a difficult cohort to recruit and engage in research studies [58]. We also had a relative overrepresentation of farmers from the Eyre Peninsula enrol in this study. The perspectives shared by these participants appeared to mimic those of participants from other regions and as such in an exploratory, hypothesis generating, qualitative study of this nature it is not concerning. Future research should work to sample farmers from other regional locations around South Australia and consider any specific gendered concerns in greater depth. Nonetheless, strength still lies in this study’s unique exploration of an important issue, reflexive approach, close consultation, participant-verified transcripts and research diary, which used in combination mitigate this potential bias.

## 5. Conclusions

This study highlighted several attitudinal, structural and farm-related issues that affect farmers’ help-seeking, which have important implications for both researchers and clinicians alike. Farmers appreciate practitioners who understand farm-work practices, routines and culture, including the unpredictable nature of farming itself. Practitioners must also understand that farmers are likely to desire independence, where possible, and are equally unlikely to present to health services unless their concern has already had a significant effect on their ability to perform farm work. Farmers’ frustration with health professionals’ lack of understanding of their way of life, and the fact that this acts as a barrier to farmers’ subsequent help-seeking, likewise highlights the importance of this research and the dissemination of its results among the rural workforce and wider rural communities. Suggestions to promote workforce development in this area include: co-designing training experiences with the future health workforce including farming communities, employing rural academics with a farming background [20] to support and mentor future workforce while on rural clinical placement, short training programmes with the current rural health workforce to highlight issues from the farming consumer perspective and building locally relevant peer-delivered support programs with farmers using evidence based approaches.

## Figures and Tables

**Table 1:** Demographic Characteristics of Participants Interviewed.

Participant Characteristics	Frequency (*n*)
**Total**	15
Men	12
**Age (years)**	
Mean (±SD)	51.7 (±12.6)
**Region**	
Mid North	4
South East	4
Eyre Peninsula	7
**Farming experience (years)**	
0–19	2
20–39	10
40+	3
**Type of farm**	
Cropping	6
Sheep, cropping	6
Sheep, beef	1
Beef cattle	1
Dairy	1

## Data Availability

The data presented in this study are available within the article.
